# An application of principal component analysis to the clavicle and clavicle fixation devices

**DOI:** 10.1186/1749-799X-5-21

**Published:** 2010-03-26

**Authors:** Zubin J Daruwalla, Patrick Courtis, Clare Fitzpatrick, David Fitzpatrick, Hannan Mullett

**Affiliations:** 1 Department of Orthopaedic Surgery, Beaumont Hospital, Dublin, Republic of Ireland; 2 Department of Mechanical Engineering, University College Dublin, Republic of Ireland

## Abstract

**Background:**

Principal component analysis (PCA) enables the building of statistical shape models of bones and joints. This has been used in conjunction with computer assisted surgery in the past. However, PCA of the clavicle has not been performed. Using PCA, we present a novel method that examines the major modes of size and three-dimensional shape variation in male and female clavicles and suggests a method of grouping the clavicle into size and shape categories.

**Materials and methods:**

Twenty-one high-resolution computerized tomography scans of the clavicle were reconstructed and analyzed using a specifically developed statistical software package. After performing statistical shape analysis, PCA was applied to study the factors that account for anatomical variation.

**Results:**

The first principal component representing size accounted for 70.5 percent of anatomical variation. The addition of a further three principal components accounted for almost 87 percent. Using statistical shape analysis, clavicles in males have a greater lateral depth and are longer, wider and thicker than in females. However, the sternal angle in females is larger than in males. PCA confirmed these differences between genders but also noted that men exhibit greater variance and classified clavicles into five morphological groups.

**Discussion And Conclusions:**

This unique approach is the first that standardizes a clavicular orientation. It provides information that is useful to both, the biomedical engineer and clinician. Other applications include implant design with regard to modifying current or designing future clavicle fixation devices. Our findings support the need for further development of clavicle fixation devices and the questioning of whether gender-specific devices are necessary.

## Introduction

The selection of any orthopaedic fixation implant is driven by several factors. However, the shape of the bone involved is commonly overlooked. When selecting a clavicular implant, there are several factors that drive the decision but the morphology of the clavicle is rarely considered. Experience to date has shown that linear scaling is a dominant mode of variation in human anatomy [1]. This paper builds on geometric data and methodology presented in a previous study analyzing linear measurements [2] in order to provide detailed information relating to the modes of variation in three-dimensional (3D) shape that occur in the clavicle. It must be noted that while intramedullary and plate fixation are accepted and widely used methods of treatment for fractures of the clavicle, current clavicular implants overlook the variations in geometry of the bone. As the clavicle demonstrates a complex anatomy, it is vital to understand the variations not only in size but also shape. This allows optimization of the implant design, in turn ensuring effective fracture fixation. This is the first 3D study that examines the shape variation of the clavicle and suggests a method of grouping the clavicle into size and shape categories based on statistical shape and principal component analyses.

## Materials

Ethics approval for this study was sought and granted through the Royal College of Surgeons in Ireland Research Ethics Committee (Study No. REC 401). Fifteen fresh frozen shoulder specimens previously used for a shoulder course and consented for research purposes were scanned using high-resolution (0.625 mm) computerized tomography (CT). These specimens were stored in a freezer compartment in airtight bags for two months after the course and defrosted for 24 hours prior to being scanned. Surrounding soft tissue was not removed. One clavicle was found to be fractured, and five were incomplete so were excluded. A further 16 high-resolution CT scans of the clavicle were obtained by searching the hospital database but four were excluded because they did not include either the superior or inferior medial or lateral aspects completely. In order to ensure none of the clavicles had pathology, search criteria included patients who had a CT scan performed for imaging of the proximal humerus or scapula. The study comprised a total of 21 clavicles.

Six of the scans were from males and 15 from females, with an average age of 54 (range 20-85 years). Twelve were from the left side and nine from the right. Biodata was available in all cases and cause of death in the group of fresh frozen specimens was known. None of the 21 clavicles scanned showed signs of a previous fracture.

All CT scans were reconstructed using Mimics software (Materialise b.v., Leuven, Belgium). These images were subsequently imported as three-dimensional (3D) STL files into Arthron, a statistical software package specifically developed by the Department of Mechanical Engineering in the institution where our research was being conducted.

## Methods

### Clavicular Coordinate Frame

**Figure 1:**
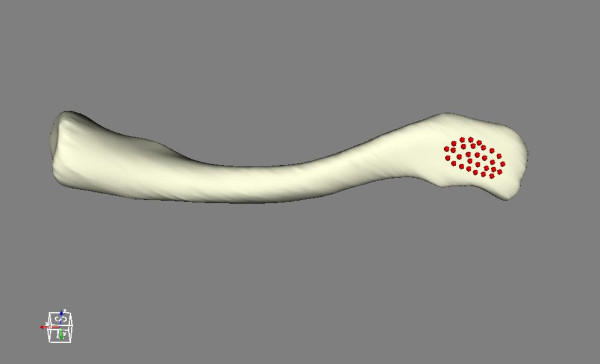
**Superolateral surface of the clavicle**. Multiple points on the superolateral flattest surface of the clavicle.

**Figure 2:**
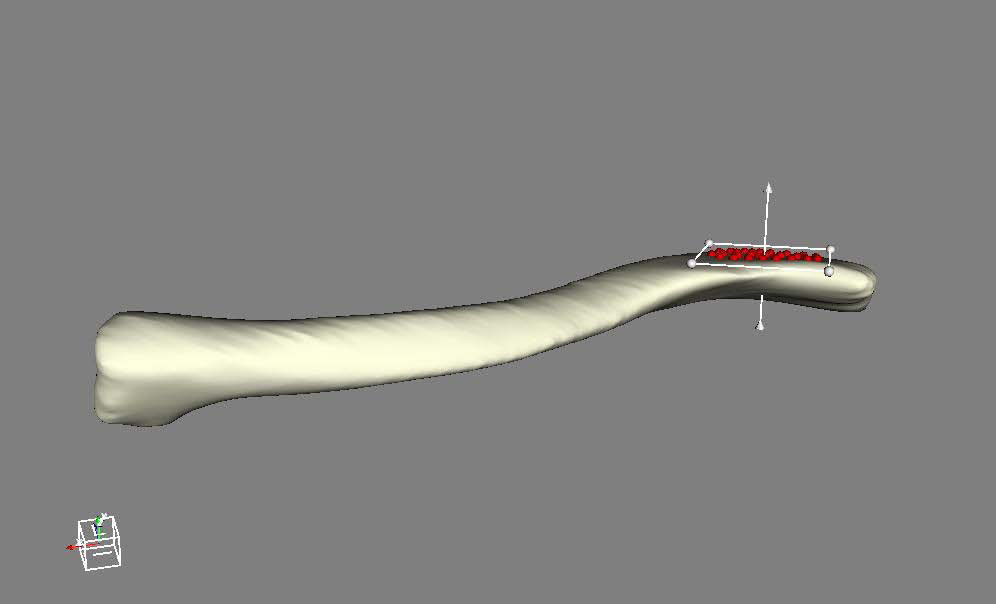
**Best fit plane**. Definition of a best fit plane.

**Figure 3:**
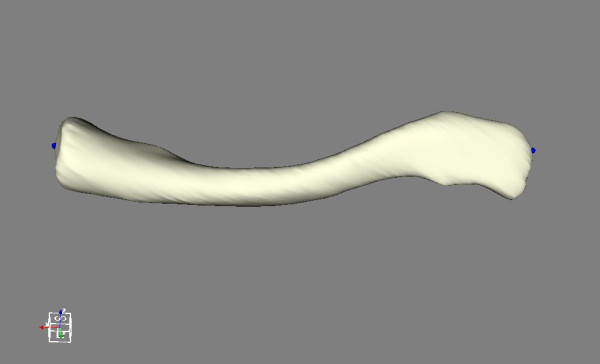
**Start and end points**. Medial and lateral edges as start and end points for slicing marked by dots.

**Figure 4:**
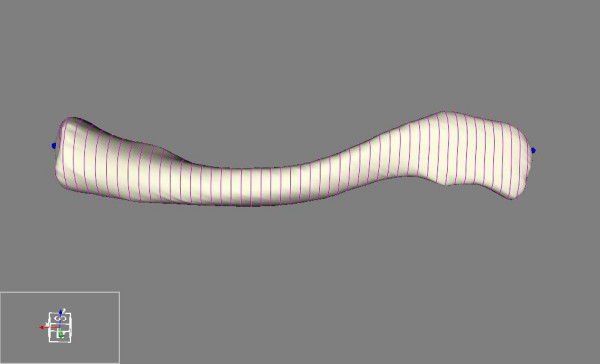
**Slicing**. Equal slicing of the clavicle.

**Figure 5:**
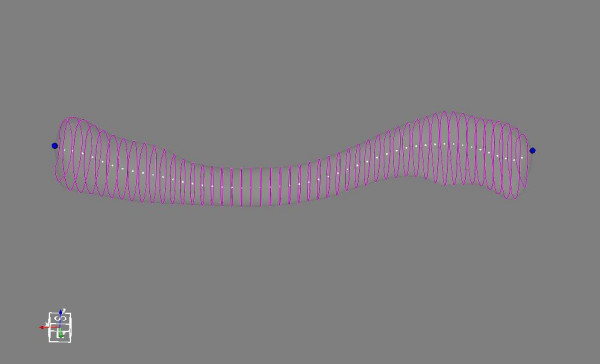
**Opacity**. Opacity reduced to show centres of each slice.

The coordinate systems of the STL files were in the original coordinate frame of the CT scanner. This was redefined using Arthron and applied to all the files. As previously described [2], multiple points on the superolateral flattest surface of the clavicle were selected (Figure 1). A best fit plane was then defined to fit these points (Figure 2). Two points representing the medial and lateral edges were then selected as start and end points (Figure 3). Between these, 50 equally spaced slices perpendicular to the line joining the two points were created (Figure 4). A best fit axis was then defined to fit the centres of these slices (Figure 5). Applying a transformation based on these axes, a coordinate frame with x-, y- and z-axes was defined. Several linear measurements including clavicle length, width, thickness as well as acromial and sternal angles were obtained using the local coordinate frame [2]. These measurements later assisted in describing the principal components of our clavicle data with the acromial and sternal angles referring to the lateral and medial angles described and referenced above [2], respectively.

### Statistical Shape Modelling of the Clavicle

**Figure 6:**
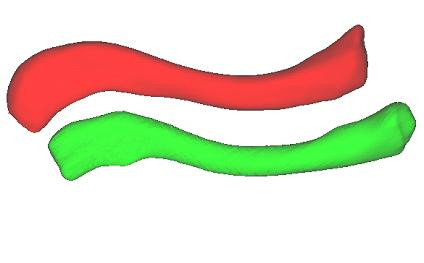
**Clavicle models**. Source and target clavicle models.

**Figure 7:**
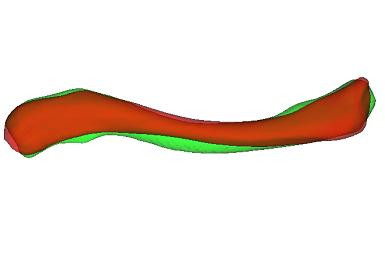
**Registration of source and target models**. Registration of source and target models using aligned local coordinate frames followed by affine ICP transformation.

Corresponding surface landmarks were established by mapping points on the surface of one clavicle onto the surface of each remaining clavicle in the study. First, the origin and axes of the above mentioned local coordinate frames of each clavicle were aligned. This methodology was found to be reproducible in assessment by both the same as well as different users. A set of sparse points, acting as anatomical landmarks, was then defined on one clavicle and an affine Iterative Closest Point (ICP) transformation using specifically developed software in Visualization Toolkit (VTK, Kitware Inc., New York, USA) was used to register the points with each of the remaining subject models (Figure 6, Figure 7). The closest points to each of the registered surface points were used to generate corresponding anatomical landmarks on each subject model.

Using the corresponding surface landmarks, a statistical model of clavicle form was generated using Point Distribution Modelling (PDM) [3]. The PDM technique represents a training set of landmark data using the mean landmarks and a set of eigenvectors which represent the linearly independent modes of variation (principal components) of the data set. Landmark data from the training set can be approximated using the eigenvectors corresponding to the largest eigenvalues *λ*_*i*_. New models can also be generated by transforming the mean shape using the linearly scaled combinations of the most significant eigenvectors. By applying a scaling limit of  the shapes generated will be similar to those in the original training set. Unlike the approach taken by Cooper et al [3], the subject models were not normalised by size hence the PDM included both size and shape variation.

Results of the principal component analysis (PCA) comprised of size and shape components. A size component reflects the variation in dimensions purely due to size, with the ratios between dimensions remaining constant while the actual values of the dimensions change. This is identifiable as a principal component (PC) whose coefficients are of the same sign and similar magnitude. Other PCs show variation in the shape of the clavicle which is due to changing ratios between dimensions, irrespective of size. Two clavicles are defined to be the same shape if scaling, rotating and translating allows them to occupy the same space.

### Cluster Analysis

Cluster analysis is a technique used to categorize objects into groups that share similar characteristics. Using the k-means function from the MATLAB^® ^Statistics Toolbox [4], the clavicles were sorted into groups based on their PC values. The correct numbers of clusters were determined by iterively varying k until the sum of the mean Euclidean distance between each data point and the centroid of the neighbouring clusters was maximized. Local minima were avoided by performing the clustering procedure with several thousand replicates.

## Results

**Figure 8:**
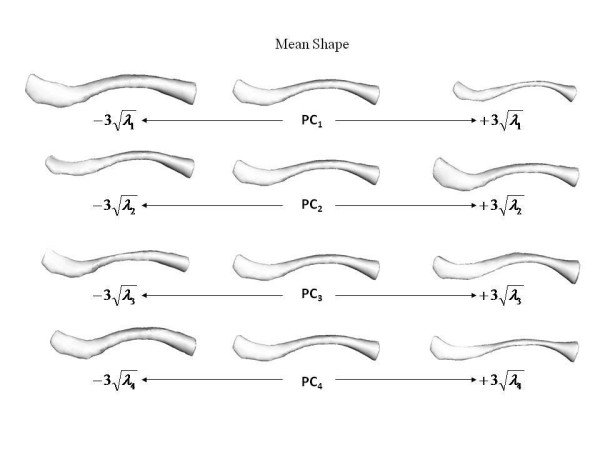
**Superior view of varying effects of principal components**. Superior view of effects of varying the first four principal components of the clavicle shape model individually.

**Figure 9:**
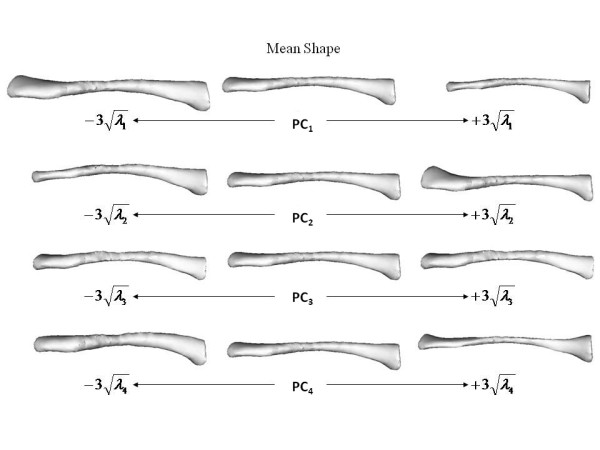
**Dorsal view of varying effects of principal components**. Dorsal view of effects of varying the first four principal components of the clavicle shape model individually.

**Figure 10:**
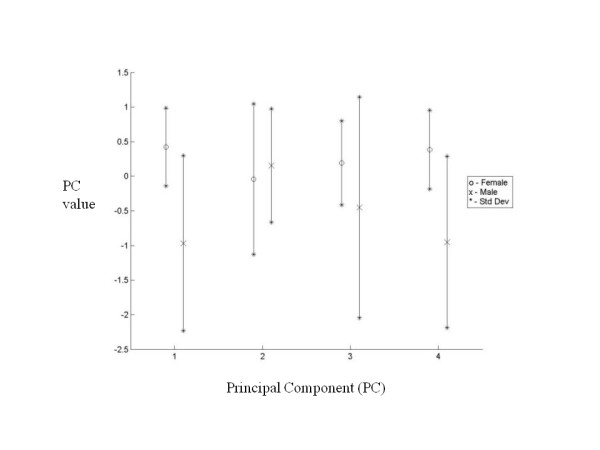
**Comparison of principal components**. Comparison of principal components showing range of values between genders.

The mean and standard deviations of the linear measurements are illustrated below (Table 1). To simplify the presentation of results, it should be noted that diameter in table 1 refers to the mean of the width and thickness measurements at the stated percentage intervals. Table 2 shows the relationship between the first four principal components and linear measurements. These relationships are visually represented below (Figure 8, Figure 9). The first principal component (PC1) reflects the variation in clavicular length as well as width and thickness at the midpoint. In our study, this represented 70.5 percent of the variation. Including the variation in lateral depth and angle dimensions (PC2) defined and described using statistical shape analysis [2], PC1 and PC2 in combination accounted for 77.2 percent of variation in dimensions. This increased to 82.2 and 86.4 percent with the addition of PC3 and PC4 which represented the variations in medial depth and angle, and width and thickness dimensions respectively. Finally, each clavicle was approximated as a linear combination of the four PCs. The range of PC values between genders is depicted below (Figure 10). By analyzing these, differences are noted between genders, most obviously in relation to PC1 and PC4.

**Table 1:** Mean and standard deviations of linear measurements.

	Length/mm		10% Diameter/mm		50% Diameter/mm		90% Diameter/mm		Sternal Angle/°		Acromial Angle/°	
	Male	Female	Male	Female	Male	Female	Male	Female	Male	Female	Male	Female
Mean	152.87	142.17	19.24	17.16	12.12	9.18	18.37	14.73	20.09	23.05	22.33	23.72
Std. Dev.	9.12	4.41	1.93	2.09	0.76	0.37	2.59	1.76	3.43	3.89	6.02	5.47

**Table 2:** Relationship between principal components and linear measurements.

	PC_1_	PC_2_	PC_3_	PC_4_
Length	*-0.99	-0.07	-0.06	0.01
10% Diameter	-0.38	-0.07	0.2	*-0.45
50% Diameter	*-0.55	0.07	-0.09	*-0.73
90% Diameter	-0.29	0.41	-0.31	*-0.46
Sternal Angle/Depth	0.37	0.13	*0.51	-0.12
Acromial Angle/Depth	0.32	*0.49	-0.28	0.08
**% Variation**	**70.5**	**6.7**	**5**	4.2

Statistically significant correlations (p < 0.05) indicated with *

**Figure 11:**
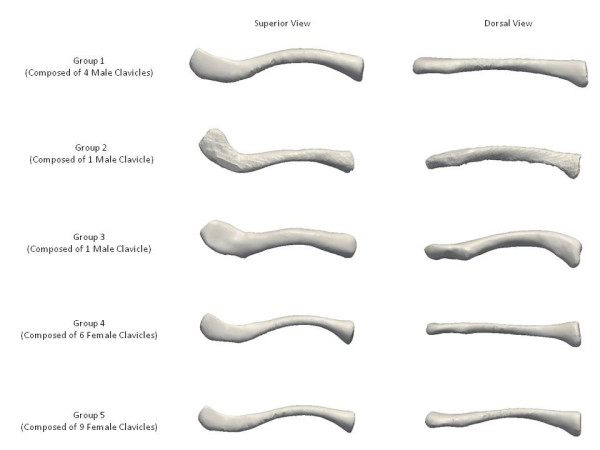
**Morphological clavicle groups**. Mean shapes of the five morphological groups of clavicles.

Using k-means clustering, clustering on a size basis using PC1 resulted in two groups. The first included five male clavicles and the second included all the female clavicles and the remaining single male clavicle. Clustering on a size and shape basis using all four PCs resulted in five groups. The first of these groups included four male clavicles, the second and third groups included a single male clavicle and the fourth and fifth groups included six and nine female clavicles respectively. The mean shape of each of these groups is illustrated below (Figure 11).

## Discussion and Conclusions

The application of principal component analysis (PCA) allows the building of statistical shape models of bones and joints. This has been used in conjunction with computer assisted surgery in the past, examples including the femur [5] and knee [6]. However, PCA of the clavicle has not been performed.

Using PCA, interrelated variables are separated into sets of linearly independent equations [7]. As no statistically significant differences were observed between principal components when comparing sides, our study focused purely on gender-specific differences. By analyzing PC values between men and women, it is clearly seen that PC1 and PC4 are gender-related. The difference in the mean values of these PCs indicates that men generally have longer clavicles that are thicker and wider at their midpoints. These features are also found to demonstrate greater variance in men. PC3, which represents the sternal depth and angle, also indicates gender-related differences with men again exhibiting greater variance. Less significant gender-related difference was noted in PC2, which represents the acromial depth and angle.

By using k-means clustering, the clavicles were also grouped on a size basis using PC1 and on a size and shape basis with all four PCs. The silhouette value [8] of a clustered data point is a measure of how similar that point is to points in its own cluster compared to points in other clusters. The optimal number of clusters was determined by varying k so the mean silhouette value of the clustered data was minimized. Unlike a study in 2008 which stated that three types of modern human clavicles exist [9], our k-means clustering results suggest the possibility of at least five morphological groups, each composed solely of a single gender. However, it must be stated that our findings were based on a limited number of clavicles and that an increased number would be more desirable in order to support the presence of the five morphological groups we describe.

**Figure 12:**
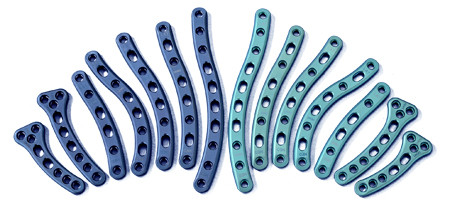
**Examples of clavicle fixation plates**. Example of a full range in size and shape of clavicle fixation plates.

**Figure 13:**
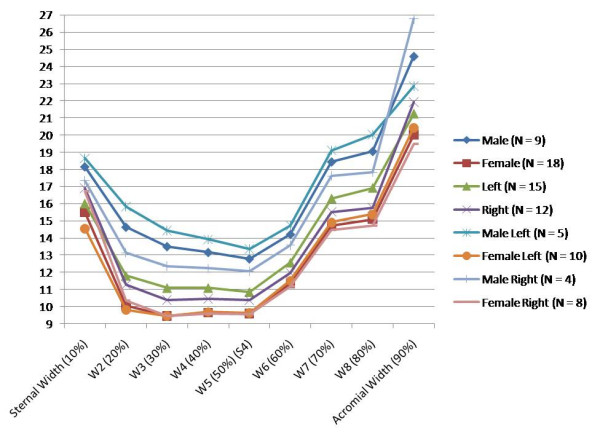
**Variation in clavicular width**. Clavicular width (mm) measured at 10% intervals of total length from sternal end.

**Figure 14:**
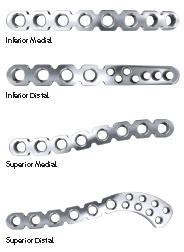
**Example of full fixation plates**. Example of a full but lesser range in size and shape of clavicle fixation plates.

**Figure 15:**
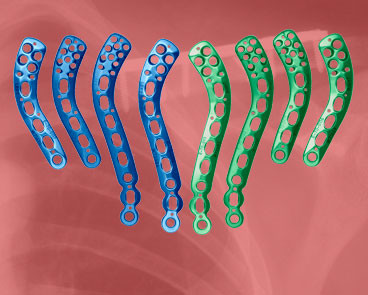
**Latest generation of clavicle plates**. Example of a newly developed range of clavicle fixation plates. Note the added curvature of the implants in addition to the curve in the plane of the natural s-shape.

In our study, 70.5 percent of variation between measurements is due to differences in width and thickness at the midpoint as well as length, rather than shape. A further 6.7 percent of variation is caused by differences in the lateral depth and angle dimensions and a subsequent 5.0 percent is due to differences in the medial depth and angle dimensions. Finally, a further 4.2 percent of variation is attributed to the change in width and thickness. Although these four modes attribute to almost 87 percent of clavicular variation, a single mode attributes to 70.5 percent. This, together with the gender-specific results evident using k-means clustering, raises the question of how much variation must be accounted for when designing an implant. Although current clavicle fixation devices exist in a range of sizes and shapes (Figure 12), none are gender-based designs. Neither do the widths of current plates vary along their length in order to closer fit the anatomic width variation of clavicles (Figure 13), something previously studied [2]. And while many plates are pre-contoured to match the natural s-shaped curve of the clavicle, they are only pre-contoured in this single plane (Figure 12, Figure 14) and do not take into account the other curvatures or bowings of the clavicle (Figure 11). While a larger sample size is always more desirable and was limited in our study secondary to the availability of cadaveric clavicles, our findings support two issues that need addressing. Firstly, the need for further research with regard to the development of variable-shape as well as gender-specific clavicle fixation devices. Perhaps more specifically for men, who as previously mentioned, demonstrate a much larger range of clavicle sizes and shapes. Secondly, and more importantly, varying the width across the entire length of clavicle plates and pre-contouring them in more than one plane would improve anatomic fit and strength of the construct, these findings clearly supporting the soon-to-be-launched third generation clavicle fixation plates by Acumed (Figure 15).

## Competing interests

The authors declare that they have no competing interests.

## Authors' contributions

ZD designed the study and is primary author who performed the majority of the research. PC performed statistical analysis and co-authored the manuscript. CF developed the statistical software package in order to perform shape analysis. DF assisted in the design of the study, supervised the research, edited and evaluated the manuscript. HM assisted in the design of the study, supervised the research, edited and evaluated the manuscript and provided clinical relevance and guidance for the study. All authors read and approved the final manuscript.

## References

[B1] Fitzpatrick C Fitzpatrick D Auger D Lee J A tibial-based coordinate system for three-dimensional data Knee 2007 14 2 133 137 10.1016/j.knee.2006.11.001 17182247

[B2] Daruwalla ZJ Courtis P Fitzpatrick C Fitzpatrick D Mullett H Anatomic Variation of the Clavicle. A Novel Three-Dimensional Study Clin Anat 2010 23 2 199 209 20069642 10.1002/ca.20924

[B3] Cooper DH Cootes TF Taylor CJ Graham J Active shape models - their training and application Computer Vision and Image Understanding 1995 61 1 38 59 10.1006/cviu.1995.1004

[B4] MathWorks Natick, Massachusetts 2007

[B5] Fleute M Lavallee S Bulding a complete surface model from sparse data using statistical shape models: Applications to computer assisted knee surgery Medical Image Computing and Computer-Assisted Intervention 1998 1496 879 887

[B6] Stindel E Briard J Merloz P Plaweski S Dubrana F Lefevre C Troccaz J Bone morphing: 3d morphological data for total knee arthroplasty Computer Aided Surgery 2002 7 3 156 168 10.3109/10929080209146026 12362376

[B7] Jolliffe IT Principal Component Analysis New York, Springer

[B8] Kaufman L Rousseeuw PJ Finding Groups in Data: An Introduction to Cluster Analysis 1990 Hoboken, NJ: John Wiley & Sons, Inc 1986

[B9] Voisin JL The Omo I clavicle: Archaic or modern? J Hum Evol 2008 55 3 438 43 10.1016/j.jhevol.2008.06.001 18692220

